# ﻿First record of the genus *Pseudaeginella* Mayer, 1890 (Crustacea, Amphipoda, Caprellidae) with a new species from Korean waters

**DOI:** 10.3897/zookeys.1169.105901

**Published:** 2023-07-13

**Authors:** So-Yeon Shin, Chang-Mok Lee, Jun-Haeng Heo, Young-Hyo Kim

**Affiliations:** 1 Department of Biological Sciences, Dankook University, 31116, Cheonan, Republic of Korea Dankook University Cheonan Republic of Korea; 2 Hanmin High School, 10955, Paju, Republic of Korea Hanmin High School Paju Republic of Korea; 3 Environmental Impact Assessment Team, National Institute of Ecology, 33657, Seocheon, Republic of Korea National Institute of Ecology Seocheon Republic of Korea

**Keywords:** Amphipod, caprellid, identification key, morphology, new record, *Pseudaeginellacarinaspinosa* sp. nov., skeleton shrimp, taxonomy

## Abstract

A new species of the genus *Pseudaeginella* Mayer, 1890 belonging to the family Caprellidae Leach, 1814 was collected from the South Sea in Korea. *Pseudaeginellacarinaspinosa***sp. nov.** is morphologically similar to related congeners belonging to the genera *Paradeutella* Mayer, 1890 and *Pseudaeginella*, in having dorsal projections on pereonites, triarticulate mandibular palp, small or absent molar, and uniarticulate pereopods 3 and 4. However, this new species is distinguished from its congeners by the position and size of dorsal projection. This is the first record of *Pseudaeginella* from the Northwest Pacific region, including Korea, and a key to species of the genus *Pseudaeginella* is also provided.

## ﻿Introduction

Caprellids, known as skeleton shrimp, are relatively small marine amphipods that are abundant and important members of the marine benthos. They inhabit a wide variety of substrates such as algae, hydrozoans, bryozoans, sponges, seagrasses, gorgonians, sediment, and other marine invertebrates (Guerra-García 2001; Woods 2009).

The family Caprellidae includes more than 440 species within 97 genera worldwide (Horton et al. 2023). Among the genera, *Pseudaeginella* Mayer, 1890 is morphologically similar to *Paradeutella* Mayer, 1890 in having the following characteristics: (1) head with a dorsal projection; (2) mandibular palp triarticulate; (3) molar small or absent; (4) pereopods 3 and 4 uniarticulate (Stebbing 1888). In their study, Iwasa-Arai et al. (2019) proposed the phylogenetic analysis of *Pseudaeginella* and the two *Paradeutella* species [*P.multispinosa* (Schellenberg, 1928), *P.tanzaniensis* (Guerra-García, 2001)] and mentioned that both genera are morphologically similar to each other. Therefore, both genera could be re-established or synonymized in the future (Zeina and Guerra-García 2016).

*Pseudaeginella* is characterized by (1) head with a dorsal projection; (2) flagellum of antenna biarticulate, swimming setae absent; (3) molar very small or absent; (4) mandibular palp triarticulate, setal formula of 1-x-1; (5) gnathopod 1, dactylus serrated distally; (6) gills present on pereonites 3 and 4; (7) pereopods 3 and 4 uniarticulate, vestigial; and (8) pereopods 5–7 6-articulate.

To date, *Pseudaeginella* comprises 13 described species (Horton et al. 2023) and is distributed worldwide, but mainly in tropical and subtropical regions (Iwasa-Arai et al. 2019). Each species in the genus is recorded in the following areas: (1) Pacific Ocean: *P.biscaynensis* (McCain, 1968), *P.campbellensis* Guerra-García, 2003, *P.polynesica* (Müller, 1990), *P.telukrimau* Lim, Azman, Takeuchi & Othman, 2017; (2) Atlantic Ocean: *P.arraialensis* Ros, Lacerda & Guerra-García, 2017, *P.biscaynensis*, *P.colombiensis* Guerra-García, Krapp-Schickel & Müller, 2006, *P.freirei* Siqueira & Iwasa-Arai, 2019, *P.montoucheti* (Quitete, 1971), *P.tristanensis* (Stebbing, 1888); and (3) Indian Ocean: *P.biscaynensis*, *P.hormozensis* Momtazi & Sari, 2013, *P.inae* Krapp-Schickel & Guerra-García, 2005, *P.polynesica*, *P.sanctipauli* Laubitz, 1995, *P.tristanensis*, *P.vaderi* Guerra-García, 2004 (Iwasa-Arai et al. 2019).

In this article, we provide a full description of the new species of *Pseudaeginella* from Korean waters, with a brief description of the female, focusing on sexually dimorphic characters. So far, 10 genera of caprellids have been recorded in Korean waters (Kim and Lee 1975; Lee 1988; Lee and Hong 2009, 2010; Hong et al. 2015; Heo et al. 2016; Shin et al. 2021). *Pseudaeginella* is newly recorded for the Korean caprellid fauna and is the first record of the genus from the Northwest Pacific region, including Korea.

## ﻿Material and method

**Figure 1.**
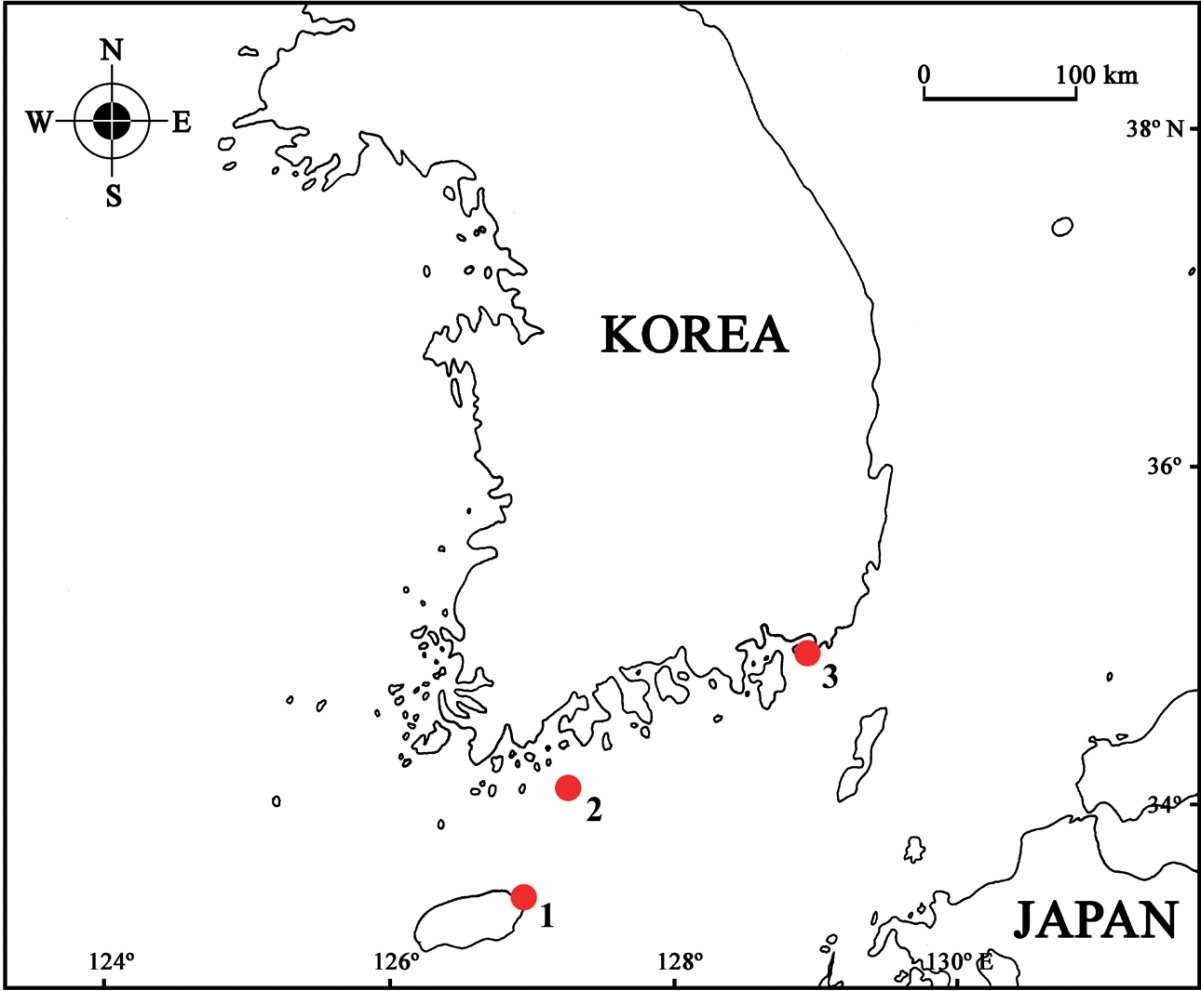
Distribution of *Pseudaeginellacarinaspinosa* sp. nov. from Korean waters (**1** Jongdal-ri, Gujwa-eup, Jeju-si, Jeju-Island, Korea **2** Geomun-Island, Geomun-ri, Samsan-myeon, Yeosu-si, Jeollanam-do, Korea **3** Namhyeongje-Island, Dadae-dong, Saha-gu, Busan, Korea).

Specimens were collected by SCUBA diving from algae such as *Ecklonia* sp. and *Sargassum* sp. in the shallow water areas of Geomun-Island, Namhyeongje-Island, Jeju-Island located off the south coast of Korea (Fig. 1). The specimens were fixed with 95% ethanol and dissected in glycerol on Cobb’s aluminum hollow slides. Permanent mounts were made using polyvinyl lactophenol with lignin pink added. Pencil drawing and measurements were performed with the aid of drawing tube, mounted on an SZX 12 stereomicroscope (Olympus, Japan) and a BX 51 interference contrast compound microscope (Olympus, Japan). Line drawings were produced using the program Clip Studio Paint (Celsys, Japan). Type specimens are deposited at the National Institute of Biological Resources (**NIBR**), Incheon, Korea and the Department of Biological Sciences, Dankook University (**DKU**), Cheonan, Korea.

## ﻿Taxonomy

### ﻿Order Amphipoda Latreille, 1816

**Family Caprellidae Leach, 1814**

**Subfamily Caprellinae Leach, 1814**

#### ﻿Genus Pseudaeginella

Taxon classificationAnimaliaAmphipodaCaprellidae

Mayer, 1890

79771B3E-8D9E-59E4-B3FF-D496BB439D0E

##### Type species.

*Pseudaeginellatristanensis* (Stebbing, 1888).

##### Diagnosis.

Head with a dorsal projection; antenna 2, flagellum biarticulate, swimming setae absent; mandibular palp triarticulate, molar absent or vestigial, setal formula of 1-x-1; maxilliped, outer plate broader than inner plate; gnathopod 1, dactylus usually bifid; pereopods 3–4 present, uniarticulate, vestigial; pereopods 5–7 6-articulate; abdomen without appendage.

##### Species composition.

The genus contains 13 species, *P.arraialensis* Ros, Lacerda & Guerra-García, 2017, *P.biscaynensis* (McCain, 1968), *P.campbellensis* Guerra-García, 2003, *P.colombiensis* Guerra-García, Krapp-Schickel & Müller, 2006, *P.freirei* Siqueira & Iwasa-Arai, 2019, *P.hormozensis* Momtazi & Sari, 2013, *P.inae* Krapp-Schickel & Guerra-García, 2005, *P.montoucheti* (Quitete, 1971), *P.polynesica* (Müller, 1990), *P.sanctipauli* Laubitz, 1995, *P.telukrimau* Lim, Azman, Takeuchi & Othman, 2017, *P.tristanensis* (Stebbing, 1888), *P.vaderi* Guerra-García, 2004.

#### ﻿ Pseudaeginella carinaspinosa sp. nov.

Taxon classificationAnimaliaAmphipodaCaprellidae

2AFC158A-FEF9-5E70-B6E5-2DCC7E99D3A9

https://zoobank.org/73AE96B5-7084-4145-B655-890AE6400ECF

**Figure 2.**
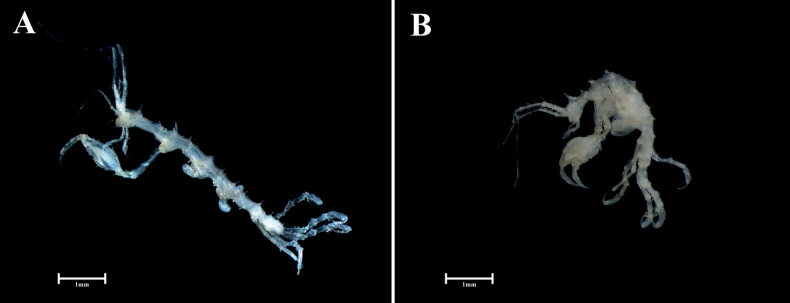
*Pseudaeginellacarinaspinosa* sp. nov. **A** male, 4.1 mm **B** immature female, 3.4 mm.

**Figure 3.**
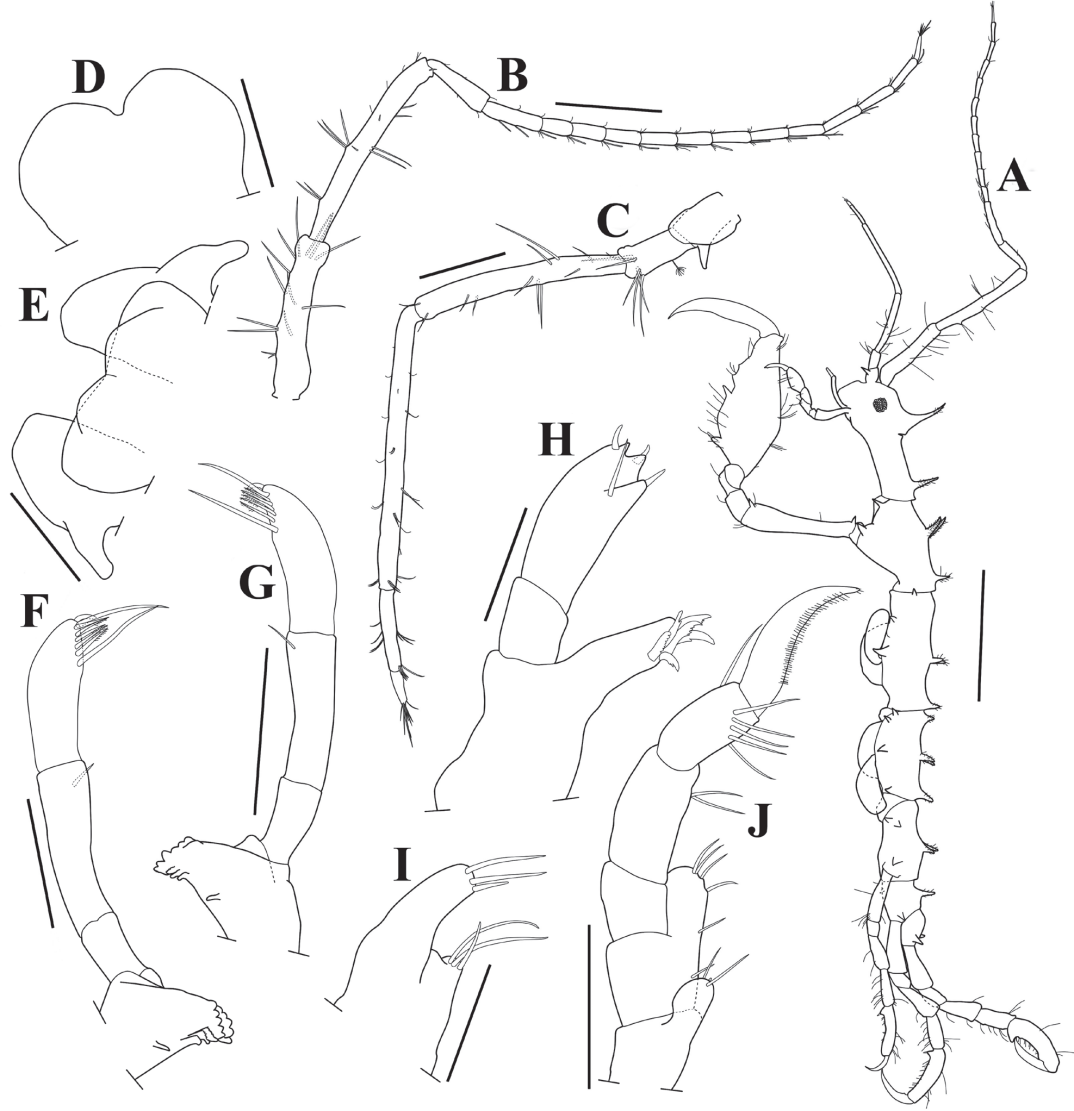
*Pseudaeginellacarinaspinosa* sp. nov., holotype, male, 4.1 mm, Geomun-Island, Geomun-ri, Samsan-myeon, Yeosu-si, Jeollanam-do, Korea **A** habitus, lateral view **B** antenna 1 **C** antenna 2 **D** upper lip **F** left mandible **G** right mandible **H** maxilla 1 **I** maxilla 2 **J** maxilliped. Male, 4.2 mm, Namhyeongje-Island, Dadae-dong, Saha-gu, Busan, Korea **E** lower lip. Scale bars: 1.0 mm (**A**), 0.2 mm (**B, C**), 0.1 mm (**F, G, J**), 0.05 mm (**D, E, H**).

**Figure 4.**
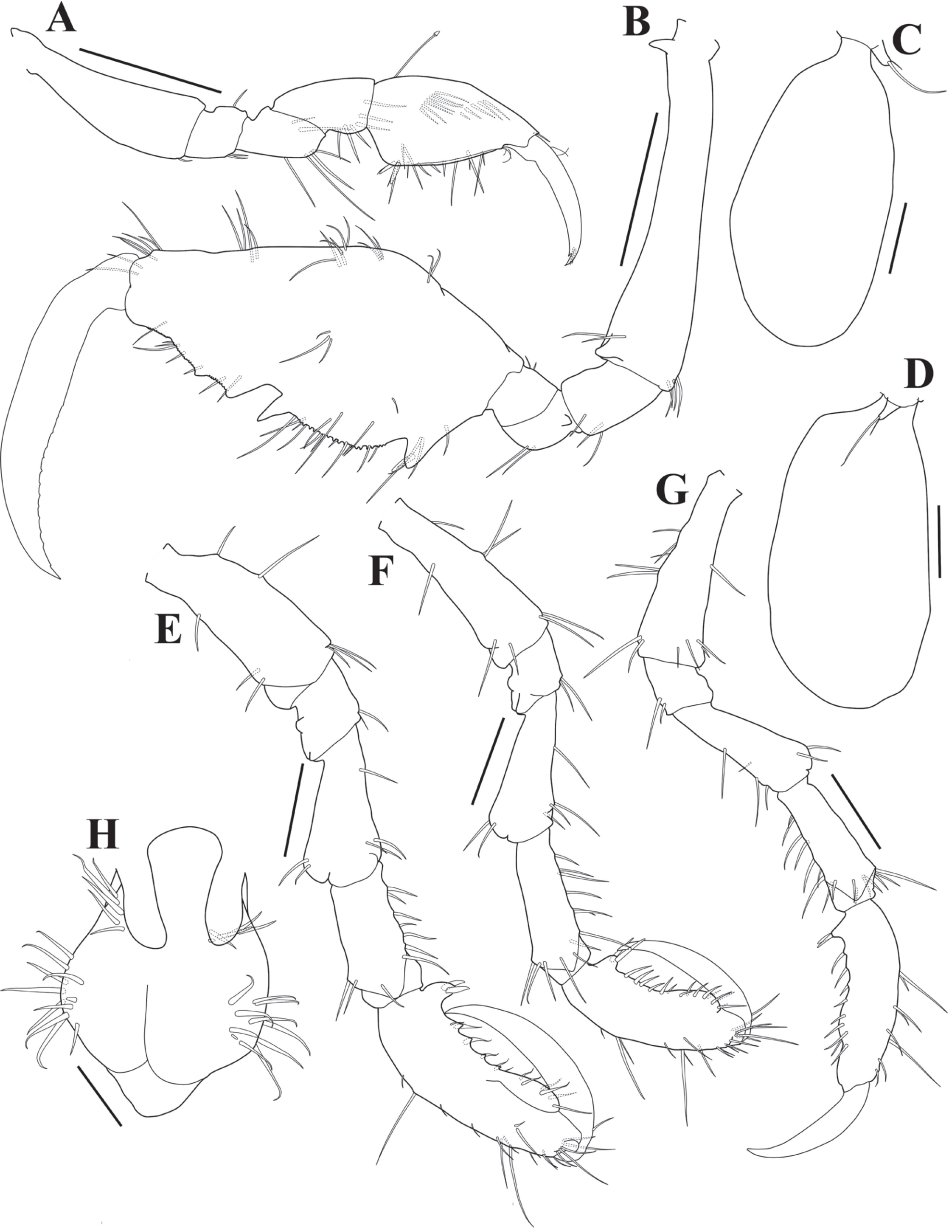
*Pseudaeginellacarinaspinosa* sp. nov., holotype, male, 4.1 mm, Geomun-Island, Geomun-ri, Samsan-myeon, Yeosu-si, Jeollanam-do, Korea **A** gnathopod 1 **B** gnathopod 2 **C** gill 3 and pereopod 3 **D** gill 4 and pereopod 4 **E** pereopod 5 **F** pereopod 6 **G** pereopod 7 **H** abdomen, ventral view. Scale bars: 0.4 mm (**B**); 0.2 mm (**A, E–G**); 0.1 mm (**C, D**); 0.05 mm (**H**).

**Figure 5.**
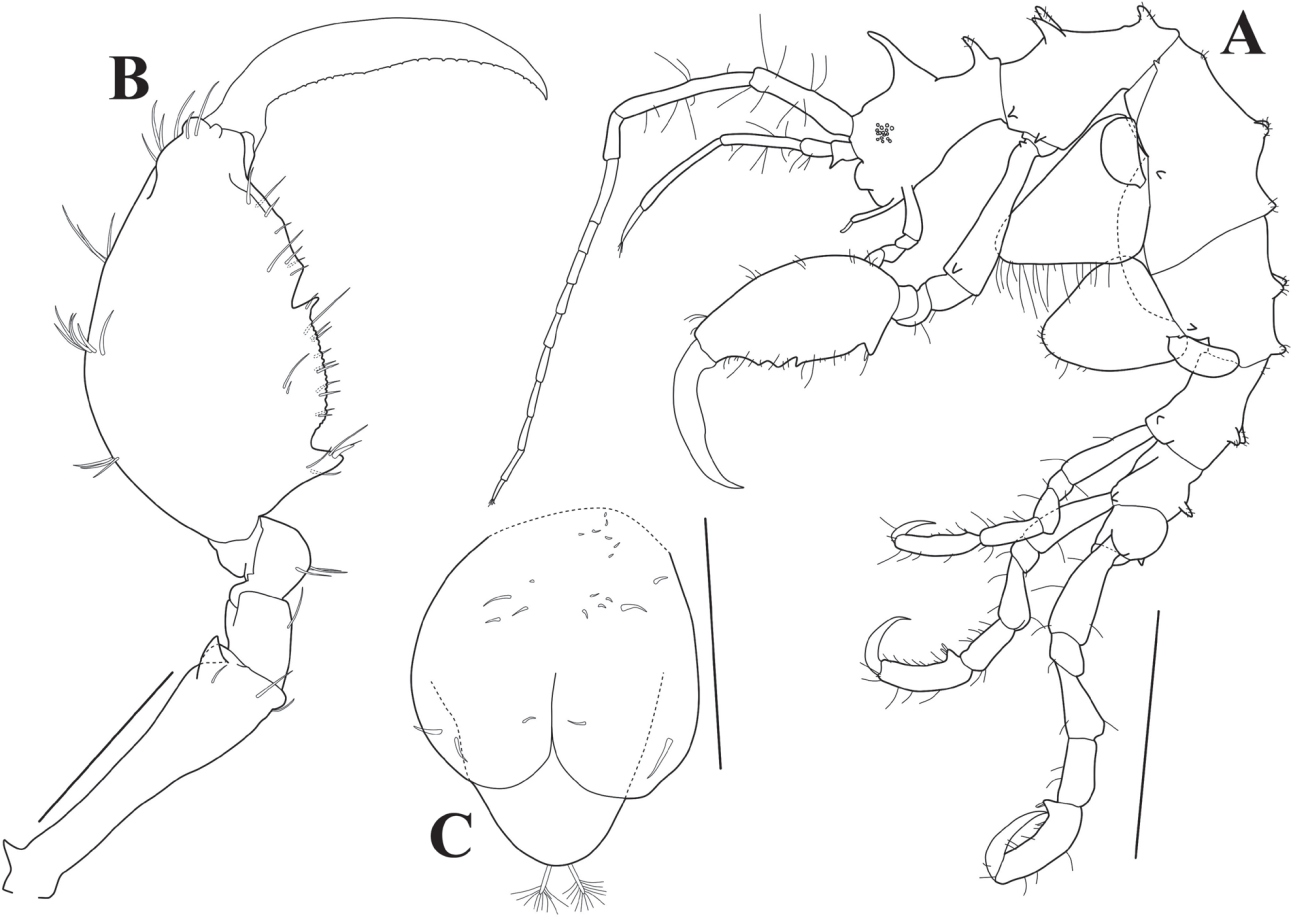
*Pseudaeginellacarinaspinosa* sp. nov., paratype, female, 3.4 mm, Geomun-Island, Geomun-ri, Samsan-myeon, Yeosu-si, Jeollanam-do, Korea. **A** habitus, lateral view **B** gnathopod 2 **C** abdomen, ventral view. Scale bars: 1.0 mm (**A**); 0.4 mm (**B**); 0.05 mm (**C**).

Figs 2 , 3 , 4 , 5 Korean name: Deung-ga-si-min-dung-bae-ba-da-dae-beol-re, new

##### Type material.

***Holotype***, male, 4.1 mm, NIBRIV0000895338, South Korea: Geomun-Island, Geomun-ri, Samsan-myeon, Yeosu-si, Jeollanam-do, 34°01'08"N, 127°18'27"E, collected from SCUBA diving, depth 7 m, Y.C. Park, 5 November 2015. ***Paratype***, female, 3.4 mm, NIBRIV0000904522 and 5 males, 5 females, DKUAMP202301, same data as for holotype.

##### Additional material.

1 male, Jongdal-ri, Gujwa-eup, Jeju-si, Jeju-Island, 33°29'29"N, 126°54'47"E, collected from SCUBA diving, depth 3 m, Y.C. Park, 5 October 2015; 2 males, 1 female, Jongdal-ri, Gujwa-eup, Jeju-si, Jeju-Island, 33°29'29"N, 126°54'47"E, collected from SCUBA diving, depth 3 m, Y.C. Park, 7 October 2015; 2 males, Namhyeongje-Island, Dadae-dong, Saha-gu, Busan, 34°53'04"N, 128°57'04"E, collected from SCUBA diving, depth 10 m, Y.C. Park, 16 November 2015.

##### Description.

**Holotype**, **male**, NIBRIV0000895338.

***Body*** (Figs 2A, 3A) slender, 4.1 mm long. Head with a projection dorsally. Eye small, round. Pereonite 1 fused with head, suture not present, with a projection dorsally; pereonites 2–5 with a pair of middorsal projections, pereonites 2–4 with a posterodorsal projection, pereonites 3–5 with ventral projections, pereonite 2 with two anterolateral projections, pereonite 3 with an anterolateral projection, pereonite 6 shortest, with a middorsal projection; length ratio of pereonites 2–7 = 1.00: 1.24: 0.99: 0.77: 0.32: 0.36.

***Antenna 1*** (Fig. 3B) slender, about 0.8× body length; peduncular articles 1–2 with several setae; peduncular article 3 short; length ratio of peduncular articles 1–3 = 1.00: 1.32: 0.43; flagellum 14-articulate, subequal in length to peduncle, each article with 1 aesthetasc ventrodistally.

***Antenna 2*** (Fig. 3C) much shorter than antenna 1, exceeding the distal end of peduncular article 3; length ratio of peduncular articles 3–5 = 1.00: 3.24: 4.35; flagellum biarticulate, swimming setae absent; about 0.1× peduncular articles 3–5.

***Upper lip*** (Fig. 3D) rounded, notched midventrally.

***Lower lip*** (Fig. 3E) well developed, smooth, without setae; inner lobe bilobed slightly (drawn from 4.2mm male).

***Left mandible*** (Fig. 3F), incisor 5-toothed, lacinia mobilis 5-toothed, followed by 3 serrulate plates; molar present but reduced; palp triarticulate; article 2 about 1.6× article 1; article 3 subequal to article 2 with a distal knob and setal formula of 1-5-1.

***Right mandible*** (Fig. 3G), similar to left one, except incisor 6-toothed, followed by 1 serrulate and 2 smooth plates.

***Maxilla 1*** (Fig. 3H), inner plate absent; outer plate with 6 stout setal teeth apically; palp biarticulate, distal article with 3 apical robust setae and 1 subapical seta.

***Maxilla 2*** (Fig. 3I), inner plate with 2 long and 1 short setae apically; outer plate with 2 long and 1 short simple setae apically.

***Maxilliped*** (Fig. 3J), inner plate small, with 2 simple setae apically; outer plate much larger than inner plate, with 3 simple setae apically and 3 simple setae medially; palp 4-articulate, article 2 longest, with 2 simple setae medially, distal article falcate, with a row of setules along inner margin.

***Gnathopod 1*** (Fig. 4A) small; propodus subrectangular, palm nearly straight with unequal simple setae, defined by 2 robust (grasping) setae proximally; dactylus falcate, bifid, with tiny accessory setae distally; length ratio of 6 articles = 1.00: 0.25: 0.43: 0.48: 0.84: 0.73.

***Gnathopod 2*** (Fig. 4B) attached midanterior margin of pereonite 2; basis elongate, subequal to propodus, slightly widening distally, with pointed projection on anteroproximal margin and subpointed projection on anterodistal portion; propodus massive, convex dorsally, width 0.5× length, with a small robust (grasping) seta on proximal projection, palm irregular and serrulate, with a poison tooth mesially; dactylus falcate, inner margin weakly serrulate; length ratio of 6 articles = 1.00: 0.23: 0.18: 0.14: 1.13: 1.05.

***Pereopod 3*** (Fig. 4C) very small, rudimentary, about 0.1× gill, uniarticulate, with 2 long and short setae distally; gill elongate-ovate.

***Pereopod 4*** (Fig. 4D) similar to pereopod 3.

***Pereopod 5*** (Fig. 4E) long and normal; propodus, palm concave and serrate slightly with small setae, defined by 2 robust (grasping) setae; length ratio of 6 articles = 1.00: 0.32: 0.69: 0.60: 1.11: 1.05.

***Pereopod 6*** (Fig. 4F) similar to pereopod 5, but more slender; length ratio of 6 articles = 1.00: 0.26: 0.72: 0.73: 0.94: 0.87.

***Pereopod 7*** (Fig. 4G) similar to pereopod 6, but more slender and serrate; length ratio of 6 articles = 1.00: 0.26: 0.70: 0.70: 0.95: 0.76.

***Penes*** (Fig. 4H) elongated, situated medially, width 0.31× length.

***Abdomen*** (Fig. 4H) without appendage, with a pair of lateral lobes and a dorsal lobe; plumose setae on dorsal lobe missing.

**Paratype**, **female** (sexually dimorphic characters), NIBRIV0000904522.

***Body*** (Figs 2B, 5A) 3.4 mm long, generally as in male, but stouter than male, pereonites 3, 4 with rounded brood pouches. Gnathopod 2 (Fig. 5B) similar to that of male, but propodus more rounded than that of male. Abdomen (Fig. 5C) similar to that of male, but with a pair of plumose setae.

##### Remarks.

We compared the new species, *Pseudaeginellacarinaspinosa* sp. nov., and related species of the genus *Pseudaeginella* (Table 1). *Pseudaeginellacarinaspinosa* sp. nov. is similar to *P.biscaynensis* (McCain, 1968) and *P.montoucheti* (Quitete, 1971) in the following features: (1) head with an anteriorly curved middorsal projection; (2) antenna 2, peduncular article 2 with ventrodistal projection; and (3) pereonites 1–5 with dorsal projections. However, our new species is easily distinguished from *P.biscaynensis* and *P.montoucheti* by the following features: (1) gnathopod 2, basis with an anteroproximal projection; (2) pereonites 1–6 with strong dorsal projections; and (3) pereonite 6 with middorsal projection. *Pseudaeginellacarinaspinosa* sp. nov. is also similar to *P.colombiensis* in the following features: (1) mandibular palp, article 3 with robust setae; (2) pereopods 3 and 4 with 2 setae. However, *P.carinaspinosa* is distinguished from *P.colombiensis* by the following features: (1) pereonites 2 and 5 with middorsal projections; (2) mandibular palp, setal formula 1-5-1; and (3) gnathopod 2, basis with an anteroproximal projection.

**Table 1.** Comparison of characters of the males of *Pseudaeginella* species.

**Characters**	**Species (male)**						
	***P.arraialensis***	***P.biscaynensis***	***P.campbellensis***	***P.colombiensis***	***P.freirei***	***P.hormozensis***	***P.inae***
Body length (mm)	6.7	4.0	6.3	3.8	3.4	3.3	4.7
Pereonites 2–6, dorsal projection (except hump)	1-0-0-0-0	1-1-1-0-0	3-0-0-0-0	0-2-2-0-0	4-1-1-1-0	0-0-0-0-0	0-0-0-0-0
Gnathopod 2, proximal projection of basis	x	x	o	x	x	x	x
Pereopods 3 and 4, # of setae	2	1	2	2	2	2	1
Setal formula of mandible	1-7-1 (left) 1-8-1 (right)	1-5-1	1-10-1	1-3	1-6-1 (left) 1-4-1 (right)	1-4-1	1-6-1
Distribution	Brazil; Gulf of Mexico	Barbuda; Bermuda; Brazil; Florida; Papua New Guinea, Australia; Saint Lucia; Tanzania; Tortugas	New Zealand	Colombia	Brazil	Gulf of Oman; Persian Gulf	Indonesia
References	Ros, Lacerda and Guerra-García 2017; Winfield and Guerra-García 2020	Gable and Lazo-Wasem 1987; Guerra-García 2002, 2004; McCain 1968	Guerra-García 2003	Guerra-García Krapp-Schickel and Müller 2006	Iwasa-Arai et al. 2019	Momtazi and Sari 2013	Krapp-Schickel and Guerra-García 2005
**Characters**	**Species (male)**						
	***P.montoucheti***	***P.polynesica***	***P.sanctipauli***	***P.telukrimau***	***P.tristanensis***	***P.vaderi***	***P.carinaspinosa* sp. nov.**
Body length (mm)	3.2	3.6	3.6	3.2	3.5	5.8	4.1
Pereonites 2–6, dorsal projection (except hump)	3-3-3-2-0	2-0-0-0-0	3-3-2-2-2	3-3-4-2-1	2-2-2-1-1	0-0-0-0-0	3-3-4-2-1
Gnathopod 2, proximal projection of basis	x	x	x	x	x	x	o
Pereopods 3 and 4, # of setae	2	1	unknown	2 (P3) 1 (P4)	1	2	2
Setal formula of mandible	1-5-1 or 1-6-1	1-5-1	1-6-1 (left) 1-7-1 (right)	1-5-1	1-5-1	1-4-1	1-5-1
Distribution	Brazil; Western South Atlantic; New Zealand	Bora Bora and Moorea, Society Islands; Seychelles	Ile Amsterdam; Saint Paul	Malaysia	Tristan da Cunha; Amsterdam Island	East Coast of Africa; Australia	Korea
References	Quitete 1971; Lacerda et al. 2011	Laubitz 1995; Müller 1990	Laubitz 1995	Lim et al. 2017	Stebbing 1888; Laubitz 1995; McCain and Steinberg 1970	Guerra-García 2004	Present study

##### Etymology.

The species name is derived from the Latin *carina* (=back) and *spinosa* (= with spinose projection) with reference to the distinct dorsal spinose projection.

##### Distribution.

South Korea (Geomun-Island, Jeju-Island, Namhyeongje-Island).

### ﻿Key to the species of *Pseudaeginella*

Modified after Iwasa-Arai et al. 2019.

**Table:** 

1	Body smooth, without projections	**2**
–	Body with dorsal and lateral projections	**4**
2	Mandibular palp, article 3 with 4 inner short setae	**3**
–	Mandibular palp, article 3 with 6 inner short setae	***P.inae* Krapp-Schickel & Guerra-García, 2005**
3	Antenna 1, flagellum 11-articulate in male, 4-articulate in female	***P.vaderi* Guerra-García, 2004**
–	Antenna 1, flagellum 8-articulate in male and female	***P.hormozensis* Momtazi & Sari, 2013**
4	Mandibular palp, article 3 with a distal robust seta	**5**
–	Mandibular palp, article 3 without distal robust seta	***P.biscaynensis* (McCain, 1968)**
5	Pereopods 3 and 4 with 2 setae	**6**
–	Pereopods 3 and 4 with 1 seta	***P.polynesica* (Müller, 1990)**
6	Pereonite 2 without anterodorsal projection	**7**
–	Pereonite 2 with anterodorsal projection	**8**
7	Pereonite 5 with middorsal projection	***P* . *carinaspinosa* sp. nov.**
–	Pereonite 5 without middorsal projection	***P.colombiensis* Guerra-García, Krapp-Schickel & Müller, 2006**
8	Pereonite 2 with 1 middorsal projection	**9**
–	Pereonite 2 with 2 middorsal projections	**11**
9	Antenna 1, flagellum 14-articulate in male, 10-articulate in female	***P.arraialensis* Ros, Lacerda & Guerra-García, 2017**
–	Antenna 1, flagellum 10-articulate in male, 12-articulate in female	**10**
10	Maxilla 2 with 4 setae on each inner and outer plates	***P.sanctipauli* Laubitz, 1995**
–	Maxilla 2 with 5 setae on each inner and outer plates	***P.tristanensis* (Stebbing, 1888)**
11	Gnathopod 2, basis longer than pereonite 2 length	**12**
–	Gnathopod 2, basis subequal to pereonite 2 length	***P.telukrimau* Lim, Azman, Takeuchi & Othman, 2017**
12	Gills as long as width	***P.campbellensis* Guerra-García, 2003**
–	Gills longer than width	**13**
13	Pereonite 3 with middorsal projection	***P.montoucheti* (Quitete, 1971)**
–	Pereonite 3 without middorsal projection	***P.freirei* Siqueira & Iwasa-Arai, 2019**

## Supplementary Material

XML Treatment for Pseudaeginella

XML Treatment for Pseudaeginella carinaspinosa

## ﻿Additional information

### Author ORCIDs

So-Yeon Shin https://orcid.org/0000-0002-1175-5447

Chang-Mok Lee https://orcid.org/0000-0002-4149-8429

Jun-Haeng Heo https://orcid.org/0000-0003-0942-7126

Young-Hyo Kim https://orcid.org/0000-0002-7698-7919

### Data availability

All of the data that support the findings of this study are available in the main text.
